# “We Don’t Want to Cry Wolf”: A Qualitative Study About Nurses’ Experiences Activating Rapid Response Teams

**DOI:** 10.1177/08445621251400541

**Published:** 2025-12-09

**Authors:** Lindsay Fitzgerald, Kim Sears, Rosemary Wilson, Lenora Duhn

**Affiliations:** 1 Perioperative Services, 71459 Kingston Health Sciences Centre, Kingston, Ontario, Canada; 2 School of Nursing | Health Quality Programs, 12363 Queen's University, Kingston, Ontario, Canada; 3 School of Nursing & DAPM, 4257 Queen's University, Kingston, Ontario, Canada; 4 School of Nursing, 12363 Queen's University, Kingston, Ontario, Canada

**Keywords:** patient safety, hospital rapid response team, qualitative approaches, nurses as subjects, escalation of care, clinical deterioration

## Abstract

**Background & Purpose:**

Patient clinical deterioration is a major safety concern. One strategy implemented for health providers to improve the timely recognition and response to patient deterioration is the Rapid Response Team (RRT). Despite this resource, patient deterioration still occurs and delayed activation of the RRT is one contributing factor. Little is known about unit-level nurses’ experiences related to RRT activation, especially within the Canadian context, which is problematic given they are the ones who are primarily responsible for initiating the process. The purpose of this study was to understand the experiences of nurses practising on general adult inpatient medicine units and their activation of the RRT.

**Methods & Procedures:**

The research question was addressed with a descriptive, exploratory qualitative study. Nurses working on general adult inpatient medicine units at an Ontario hospital study site were purposively recruited to participate. Semi-structured interviews with the six participants were held online and audio-video recorded. Inductive, thematic analysis was used.

**Results:**

Eleven themes about the barriers and facilitators to RRT activation, and one overarching theme—the *Self-Imposed Complexity of Deciding to Activate the RRT—*resulted in relation to the nuanced, multi-factorial decision-making process unit-level nurses undertake when considering activation.

**Conclusion:**

This study contributes novel information to better understand RRT activation by nurses and will inform practice changes surrounding RRT policies, nursing education about the RRT, and new research on optimizing strategies for RRTs and deteriorating patients. The multi-layered activation process intricacies positions future work to improve escalation of patient clinical deterioration.

## Background & Purpose

Patient clinical deterioration is the worsening of a patient's physical condition, thereby increasing their risk of critical illness or death (Al-Moteri et al., 2019; Lavoie et al., 2016). “Failure to appreciate status changes/deteriorating patients” ranked third in acute care risk in 2020 according to the Health Insurance Reciprocal of Canada (HIROC, 2020, p. 1). Failure to recognize and respond to patient deterioration is not only a problem in Canada, but globally (Al-Moteri et al., 2019; Liaw et al., 2018). Strategies have been developed to help providers detect deterioration, yet unmanaged patient deterioration still occurs (Padilla & Mayo, 2019).

The Rapid Response Team (RRT) is a 24-h, seven days a week service typically consisting of expert critical care doctors, nurses, and respiratory therapists working as a small team (the size of which can vary) and consulted for their expertise in assessment and management of deteriorating patients in non-critical care areas (Critical Care Services Ontario, n.d.). RRTs were created to address hospital mortality related to failure to recognize and rescue deteriorating patients (McNeill et al., 2019). Successful implementation of RRTs has been associated with a reduction in hospital mortality, as well as cardiopulmonary arrest rates (Al-Omari et al., 2019); however, delays in activation, resulting in longer hospitalizations and increased mortality, have been found (Bingham et al., 2018; Chua et al., 2019; Moreira et al., 2018; Padilla & Mayo, 2019; Zhang et al., 2024), as well as no association with reduction in hospital mortality (Girotra et al., 2022).

Further, in a recent national cohort study based on data of nearly 13,000 in-hospital cardiac arrest patients (IHCA) from the Swedish Registry of Cardiopulmonary Resuscitation, the researchers found that the RRT-reviewed patients had a lower unadjusted 30-day survival rate compared to those who were not seen by the RRT (Thorén et al., 2023). The authors note that those seen by the RRT were “probably severely ill” (p. 6) and also highlight delayed RRT activation (up to 24 h) was reported as frequent in the analyzed subgroup (*n* = 82). For RRT to be effective, it must be activated and consistently the research suggests delays in activation persist. Why this is so and the experiences of nurses regarding the RRT requires ongoing investigation given they are the ones who are primarily responsible for initiating it and especially within a Canadian context where there is a lack of substantive research. This understanding is an essential step toward safer practice.

The purpose of this descriptive, exploratory qualitative study was to advance what is known about the experiences of nurses and their activation RRTs. The research question was: What are the experiences and perceptions about activating a RRT as described by nurses practicing on a general adult inpatient medicine unit? The decision to focus the study on a medicine unit was based on the fact early detection of patient deterioration and activation of the RRT is particularly important on these types of settings, as patients whose condition deteriorates there “…have considerable morbidity and mortality” (Escobar et al., 2020, p. 1951).

## Literature Review

Using the databases MEDLINE and CINAHL, as well as the reference lists of selected papers, the evidence was searched regarding RRT and nurse practice. The country of origin, as based on first author (LF) location unless otherwise indicated, was primarily Australia, the United Kingdom, and the United States (US); there were three Canadian studies (two of which were by the same researchers).

### Findings

#### Enablers to RRT Activation

RRT activation is influenced by nursing staff experiences with and the perceptions about it, the tools and technologies available such as specified activation criteria, and the monitoring technology in use (Chua et al., 2017). Further, nurses state unit culture and team member characteristics, such as leadership and support from their coworkers to help them with nursing duties during a RRT call, also enable activating the RRT (Astroth et al., 2013; Bingham et al., 2020; Jenkins et al., 2015). Also, as reported by nurses, the Early Warning Score (EWS)—an aid to prompt nurses to escalate patient care, if warranted, through a scoring system for identification of acute abnormalities in physiological measurements, such as blood pressure, respiratory rate and level of consciousness (Henn et al., 2017; Lee et al., 2018)—and RRT can positively affect their competence in sensing clinical deterioration and develop knowledge, skills and self-efficacy to react to it (Jensen et al., 2018).

#### Barriers to RRT Activation

##### Inadequate Knowledge

Inadequate knowledge of the RRT has been a barrier to its use (Jenkins et al., 2015; Tilley & Spencer, 2020). Nurse participants have reported misunderstanding the purpose and function of the RRT, as well as the RRT activation criteria (Massey et al., 2014). Similarly, unclear policies, protocols and staff training about the roles, responsibilities, purpose of, and activation criteria for the RRT have been reported as barriers to its activation (Astroth et al., 2013; Chua et al., 2017; Chua et al., 2019; Moreira et al., 2018). Nurses do report “…challenges in identification of subtle changes in patient deterioration” (Ruiz et al., 2024, p. 1).

##### Negative Experiences of RRT Members and Unit Nurses

From the perspective of RRT members, it has been found they perceive an inability of unit staff to recognize and respond to clinical deterioration (Currey et al., 2018). Notably, communication and documentation, lack of knowledge, inadequate assessment skills, and lack of critical thinking, were areas of concern, as well as a deficiency of system governance (Currey et al., 2018).

From the perspective of the unit level nurses, those who valued the RRT also found it to be time-consuming as it increased their workload, and they feared criticism or being reprimanded for inappropriate activation (Chua et al., 2017; Chua et al., 2019; Currey et al., 2018; Massey et al., 2014; McGaughey et al., 2017; Menon et al., 2018; Padilla et al., 2018; Ruiz et al., 2024; Tilley & Spencer, 2020). Seeking further justification for subtle or subjective clinical changes and hesitating about activating the RRT to consider the potential reactions of the RRT members regarding the reason for the call, were other barriers nurses reported (Chua et al., 2017; Chua et al., 2019; Tilley & Spencer, 2020).

In a systematic review by Padilla et al. (2018), the evidence regarding a nurse's years of experience and the tendency to activate the RRT was contradictory­—there was evidence indicating nurses with less experience more often activated the RRT as compared to those with higher degrees of experiences and vice versa. However, based on the findings in the systematic reviews by Chua et al. (2017) and Padilla et al. (2018) and the survey study by Jenkins et al. (2015), experienced nurses activated the RRT more because they feel less intimidated by RRT team members, have greater confidence, and use intuitive clinical judgement from past experiences. Likewise, Parker (2014) found nurses who used analytical decision-making skills tended to be older and more experienced nurses and activated the RRT twice as often as intuitive decision-makers. It has been postulated junior nurses felt RRT activation increased their workload, reduced their skill set in managing deteriorating patients, and perhaps they were unsure of their clinical knowledge and felt it was unnecessary to contact the RRT (Padilla et al., 2018; Tilley & Spencer, 2020).

##### Role of Primary Physician

Some nurses feel uncertain about activating the RRT without first contacting the primary physician (Chua et al., 2017; Chua et al., 2019; Padilla et al., 2018). Other nurses contact the RRT due to a lack of support from the primary team, an inability to reach the primary physician, or because of concerns about the mismanagement of the patient (Chua et al., 2017; Chua et al., 2019; Padilla et al., 2018). Nurses also reported resistance from primary physicians when they involve RRTs, while physicians felt the RRT activation implied their inability to manage the patient, and others felt it was a lost learning opportunity for junior physicians (Chua et al., 2017).

##### Ineffective Communication of Deterioration

In the surveillance and management of deteriorating patients, the absence of effective communication can increase anxiety, and fear or concern, which can result in activation of the RRT (Martland et al., 2016). However, in contrary, physicians have identified, even with the use of a communication tool, nurses ineffectively articulated deterioration, while nurses report difficulty convincing physicians of deterioration, especially if it was more subjective than objective (Chua et al., 2019). There is the sociocultural belief nurses are to follow an interprofessional hierarchy when escalating care, of which the RRT is not considered a part (Allen et al., 2017; Bingham et al., 2020; Chua et al., 2019; Moreira et al., 2018; Tilley & Spencer, 2020).

## Summary

Nursing education, positive working relationships between unit nurses and RRT members, as well as using both the EWS and the RRT helped to achieve the desired outcomes of the RRT. Previous negative experiences and ineffective communication were barriers to activating the RRT. Early signs of deterioration were not always recognized by nurses and the lack of understanding of the purpose, function, and activation criteria by nurses continues to be a barrier to RRT activation There remain gaps in knowing why nurses delay RRT activation, particularly in the Canadian context.

## Methods and Procedures

### Methodology

For this study, a descriptive, exploratory qualitative approach was used (Kahlke, 2014; Sandelowski, 2000). *Descriptive qualitative* studies describe the fundamental information as it is (Sandelowski, 2000). Research questions aligned with this kind of approach include: “…What are peoples’ responses (e.g., thoughts, feelings, attitudes) toward an event? What reasons do people have for using or not using a service or procedure?…” (Sandelowski, 2000, p. 337). Polit and Tatano Beck (2017) state qualitative descriptive research includes questions about, “what are the dimensions or characteristics of the phenomenon?” (p. 15), with exploration as questioning, “what is really going on here?” (p. 15). Given our lack of knowledge about why nurses delay RRT activation, it was important to garner a deep understanding, and as grounded in their words and what they felt was important to voice.

## Setting and Sample

The study site was an acute care teaching hospital with a RRT, specifically in seven adult inpatient medicine units in Queen's University. The same recruitment strategy was applied across all units.

The population was licensed, practicing RNs working on general adult inpatient medicine units. Only RNs were chosen because in deteriorating patient scenarios (where there is an unpredictable outcome, complex and changing patient needs and or a risk for negative outcomes), Registered Practical Nurses (RPNs) [or Licensed Practical Nurses in other parts of Canada] are instructed to transfer care of the patient to an RN (College of Nurses of Ontario, 2025). As such, the need for an RPN to activate the RRT would be less frequent. Purposive sampling was used, and the study inclusion criteria were: 1) an RN at any stage of their career; 2) working on one of the included medicine units (notably, this included *float nurses* [those nurses who rotate each shift between units], as their involvement to safely staff the units is frequent and they are recognized as part of the unit culture); 3) nurse's previous experience with the RRT was not mandatory, as understanding the reason for not calling the RRT (as relevant) was equally important. The exclusion criterion was nursing staff who do not provide direct patient care.

Six individuals participated in this study and no participants dropped out of the study. Their mean age was 32 years (median = 29.5 years; range of 22–50 years), and their mean years of experience was 5.69 years (median = 3.75 years; range of 8 months to 13 years). Two individuals worked within the float pool, three individuals were from the same medicine unit, and one individual was aligned with three medicine units. Pseudonyms are used for participants.

## Recruitment

Once ethics approval was received, the manager (of two of the units) of the selected medicine program forwarded the study information to the nursing staff via email (the research team was not privy to staff email addresses), and recruitment posters were displayed on the units. Interested nurses were asked to contact a research team member (first author (LF)) via email. Three weeks after distribution of the original email, the manager re-sent the invitation. An additional approach was introduced after study recruitment began on the first two units; this included attendance of a research team member (Queen's University) at “huddles” (informal staff meetings) to present the study and answer any staff questions and was conducted approximately one to two times per week. Shortly after this additional approach began, the study was also opened on the additional five units, wherein the same strategy was applied. All consenting participants were entered into a draw to win a gift card. Recruitment occurred over a four-month period, which was potentially impacted given it was summer vacation time, as well as the study site was continuing to experience COVID-19 cases.

## Data Collection

Semi-structured interviews, conducted only by the first author (LF), were used as the method for data collection (Percy et al., 2015), and some pre-determined questions—as informed by the literature, (e.g., Astroth et al., 2013; Massey et al., 2014)—were used to guide the interview. The participants received the questions for review via email one week prior to their interview. The questions included demographic questions, as well as open-ended questions about their experiences related to RRT activation (e.g., describe a typical RRT activation). Interview questions were modelled from the studies by: Astroth et al. (2013), Massey et al. (2014), and Shapiro et al. (2010) and the conceptual framework “A Human Factors Framework: Contributing Factors to Adverse Events in Healthcare” by Henriksen et al. (2008). Each interview was approximately 40 to 60 min. Individual (audio and video recorded) interviews were conducted online using the program Zoom^©^. After the interviews, the participants were emailed a copy of their interview transcript, and all of them confirmed its accuracy, with no additional information provided. Additionally, once their one individual interview was complete, participants were invited to join an online focus group using Zoom^©^. The one focus group (conducted only by the first author (LF)) was an opportunity to present a summary description of the collective interviews and for participant feedback regarding the initial findings and any new information they wished to contribute. Those unable to attend were emailed the information; one person (of six) attended the focus group and three people provided some feedback via email.

## Data Analysis

Initially, the interviews were transcribed verbatim (as facilitated by the speech-to-text function on Zoom^©^) and were independently reviewed against the audiovisual files for accuracy by two members of the team (LF, LD). This was followed by mean/median calculation for the demographic variables, and thematic analysis of the transcripts. The elements of the conceptual framework were used to guide thematic analysis (e.g., coding) and to assist in probing deeper into the data but did not overshadow the participants’ accounts. The data was analyzed using an inductive, thematic analysis with a constant comparison approach, modelling the directives of Percy et al. (2015, p. 83). Starting with the first participant, the data was analyzed and continually compared to the findings of the next participant's data, then back to the previously coded data, and so on until all the participants’ data were analyzed, resulting in the continual transformation of patterns and themes (Percy et al., 2015, p. 83).

As Percy et al. (2015) describe, the analysis began by highlighting any important or meaningful phrases or paragraphs, or information from the participants’ transcripts that relates directly to the research question (p. 83). This made the data more manageable to work with and significant pieces easier to find. The organization of the data through coding occurred by first creating a coding scheme, then clustering ‘like data’ together to help to identify prominent or repeated themes, as well as the progression of any patterns developing amid these themes (Percy et al., 2015; Polit & Tatano Beck, 2017). Tables were used to organize the codes, patterns and themes. Each code was numbered on the highlighted copy of the transcript and the numbers corresponded with a code label, created by using the words of the participant (or a paraphrased version of that) from the data and corresponding code definition. After this process was complete for the first participant's data, the same steps followed for each of the following participants’ data, while constantly referring back to the previously analyzed data for comparison (Percy et al., 2015, p. 83). At this stage, LF began to take note of any visible patterns among the different codes and searched for any overarching themes, and for each theme, wrote a detailed description of it, including direct quotes from the participants to represent it most truthfully (Percy et al., 2015). The final themes and patterns were synthesized, providing concise data to help answer the research question.

This complete data analysis process (including coding) was conducted by the first author (LF) and in regular consultation with the last author (RW) and reviewed with the full team to determine resonance and clarity of meaning. Discussions among the team allowed for reflection on the descriptions and presentation of the data.

## Ethics

Ethical approval was obtained from the Queen's University and as link to the study site, specifically, Kingston General Hospital site of Kingston Health Sciences Centre (KHSC). Individuals who approached the team were contacted and the study was discussed. If interested, a date to conduct an interview was arranged. Prior to the interview, a consent form/information letter and interview questions were provided via email to each participant. Once individuals agreed, they were asked to keep a copy of the consent form/information letter and verbal consent was obtained at the start of the interview. For confidentiality purposes, all personal identifiers and proper nouns were removed from any transcripts/data analysis documents and replaced with a code, and privacy was maintained through secure storage of all research documents and videos via our university online platform and password protected computer.

## Trustworthiness

To maintain trustworthiness and integrity in the study, strategies regarding credibility, dependability, confirmability, transferability, and authenticity (Lincoln & Guba, 1985; Polit & Tatano Beck, 2017) were implemented as outlined in Table 1.

**Table 1.** Strategies for Trustworthiness.

Item	Approaches
Credibility	Credibility was addressed through approaches such as the use of well-established research methods, audio video recording, precise transcription, member checking and triangulation (e.g., new information gained from the previous interview was reframed as a question for the following the participant to garner their perspectives), peer review and constructive critiquing, and maintaining reflexive journaling, all of which are recognized strategies (Krefting, 1991; Shenton, 2004).
Dependability	Regarding dependability, documentation throughout the research process was retained, triangulation (i.e., interviews and a focus group) and member checking occurred, and there is a detailed description of the study process and findings (Shenton, 2004).
Confirmability	Based on identified strategies for confirmability (Krefting, 1991), peer debriefing about data analysis and member checking occurred. Further, to ensure accuracy, and true reflection and interpretation of the participants’ experiences, intercoder checks occurred (Polit & Tatano Beck, 2017).
Transferability	Transferability was enabled through detailed and rich description of the sample, data collection process and the findings (Shenton, 2004).
Authenticity	To facilitate authenticity, reflexive journaling, audio video recording, and verbatim interview transcribing was done, and all perspectives were included and accounted for (Polit & Tatano Beck, 2017, p. 562).

## Positionality and Reflexivity

Supported by the team and mentored by expert research faculty, the first author (LF) was involved with each phase of the research, including conducting all the interviews, the focus group, and the analysis. Given the first author (LF) undertook this study as a Masters student (and novice researcher), with over 10 years of clinical experience as a registered nurse, and works at the study site within their Rapid Assessment of Critical Events (RACE) team, initial time was taken to consider their thinking and opinions regarding the RACE team, and reflexivity during the research was critical. The study was inspired, in part, based on the first author's (LF) experiences of witnessing delayed RRT activations. Notably, three participants were aware of the first author's (LF) RRT role, but as with all participants, LF reminded them of confidentiality and encouraged a safe space that welcomed all opinions and experiences and for sharing that ‘held’ no judgement—and with a primary goal of improvement and no blame. This included reflection after each interview regarding thoughts, perspectives, beliefs, and comments about how the interview was conducted and the information. Research team discussions were frequent, allowing for attention to rigorous processes and genuine engagement with the data, through analysis and presentation.

## Results

### Main Patterns and Themes

Eleven themes were illuminated, with enabler and barrier patterns (Table 2).

**Table 2.** Study Themes.

Theme		Participants’ (verbatim) Words
*** –Enablers–***		
1	A Nursing Team Effort for RRT Activation	*Often we’ll see people consulting, kind of, other nurses on the floor, more experienced nurses…they will kind of talk amongst each other if it's not of urgent nature. **(Elizabeth)***
2	Increasing Nursing Experience and Confidence Facilitates RRT Activation	*I had already called maybe a good handful of RRTs so, I was comfortable a little bit more in my skills, I guess….but, I think, just over time you gain more intuition and knowledge, right, like, your intuition kicks in that I've probably seen this before or I've seen something like this before, and I think I might know where this is going so I'm more on alert about the patient status. (**Jessica)***
3	A (Mostly) Common Understanding of When to Activate the RRT	*I kind of just judge if it's like something out of what I can manage. **(Hannah)***
4	It Made it Easier for Me to Call the Next Time: Past (Positive) Experiences with the RRT	*They [nurses] feel supported by RRT…if they’re [nurses] ever in a situation that is uncomfortable or they’re not getting the response [by the medical team], they know RRT will be there.* (***Stacy)***
*** –Barriers–***		
5	I’m Not Sure I Ever Learned Why to Call—Lack of Education about RRT	*I feel like a refresher would be really good. Just to kind of remind people what is appropriate, what maybe is not. That kind of thing. Although I don’t know if you’d ever want to like, tell people what is not appropriate, because then they won’t, they’ll be deterred from calling…because you want to maintain, you know, that open approachable team, but you also want to be called for things that are actually RRT. **(Stacy)***
6	The Fear Factor in Activating RRT	*The first few times you call, it is nerve wracking as a new nurse because it's an emergency situation that you’re initiating…and if it's not emergent then you’re kind of left feeling like oh, what didn’t I know or what made me feel like I needed to do this…leaving the room questioning my own skills and knowledge. **(Jessica)***
7	We Don’t Want to Cry Wolf—The Unwritten Rules About When to/not to Activate the RRT	*I don’t want to call them for something silly when they’re going to be busy doing other things, but at the same time, it could not be silly too…and lead to something terrible. **(Laura)***
8	The Team Wasn’t Listening to Me—The Tension in Calling (or Not) RRT	*I kind of had suggested RRT, because it didn’t seem like they were making a lot of progress and the team was like, no, we’ve got this like we’re here, we’re working on it*. *I do feel there*'*s a sense of like ownership of the patient's care and if the team responsible for the patient feels they’re doing what's appropriate, but the nurse doesn’t, I feel like there's kind of like tension in calling RRT…it's almost like they don’t, they feel like we don’t trust them, in a way*. ***(Stacy)***
9	Calling Over the Loudspeaker to Them Coming with Their Cart—RRT Activation is Overwhelming	I *don’t know if it*'*s the whole watching RRT come in with their cart and like that overwhelming, you know, effort that it takes. **(Jessica)***
10	We’ll Just Bypass This and Go Straight to RRT—Varying Unit Cultures and Implication to the RRT	*I think there's more support during the daytime from the teams. So, possibly, I think the RRT is called a little bit less during the day because there's more staff there…there's charge nurses, there's residents’ rounds…whereas overnight, there's not as much support from the teams, the nurses have higher patient loads, so it might take the nurses slightly longer to get into see a patient and umm, recognize the change, and by that time, RRT would need to be called…so I think nurses would be quicker to call RRT on nights. **(John)***
11	Colleagues Can Hold You Back in Calling—Varied (Mis)Communication RRT Practices	*It's just like a lot of different people saying different things about this and like I just need some clarification here. **(Laura)***

(RRT = rapid response team)

#### Enablers

**Theme 1: A Nursing Team Effort for RRT Activation.** Nurses engage with each other to problem-solve as a group before calling the RRT and then work as a team once a call has been placed. Nurses reported they would rarely call the RRT without first asking the opinion and help of a colleague they trust or another nurse with more experience. Jessica said, *Oh for sure! I would have asked somebody else—well, I might ask somebody else what they think, somebody that I trust*. Further, nurses like to complete tasks, such as giving medications, collecting bloodwork, starting an intravenous, and completing thorough physical assessments, before involving the RRT. Jessica also offered, *there's always things that you can do for the patient, right…to see if I can make things a bit better before you call, which is something you want to do, because you want to make sure you’ve tried these things.* Additionally, nurses seem to naturally coordinate into different roles during patient deterioration situations and when RRT is activated. Laura stated:

once an actual code is called, our floor very generally, as a rule, is very good at like everybody hands on deck, like let's work together.…someone's always monitoring the vitals…we just kind of fall into spots, fill in where we need.

**Theme 2: Increasing Nursing Experience and Confidence Facilitates RRT Activation.** It was revealed the more years of nursing experience one has, the more confident they become in their skills and abilities. Their increased confidence and their prior experiences contribute to their comfort in activating the RRT if needed, and others seek that expertise. Hannah said, *…usually I do, since I’m so junior, I usually will consult with some more senior staff to get their opinions as well, because maybe they know something, like how to treat things, that I don’t*. Stacy shared,

Your assessment skills and your ability to pick up on when things are deteriorating to a point you need help, develop over time…so, at the beginning, you might not be able to pick up on those like, subtle changes in respiratory status and those types of things, until it's kind of too late….the more experience you have, the more you have to rely on, in terms of previous experiences…as you have more experiences, you may have used the RRT more often and become more comfortable about what is appropriate to call and what's not. My first year of nursing when I didn’t have that confidence and that, the confidence in my nursing voice yet… when I’m a new nurse, I will not have the confidence to kind of speak up over those doctors who are saying no, like, we’ve got this, whereas 11 years later, I can be like, absolutely no, I’m going to call RRT because of this, this and this. Once I learned to follow my gut, I would call RRT.

John added, *I don’t really feel a hesitation to call RRT, because, I know that*'*s why RRT exists…my job, basically is to identify something I think is abnormal…I should call you guys* [RRT]…*it has come with experience, just not worrying about what people will think*.

**Theme 3: A (Mostly) Common Understanding of When to Activate the RRT.** Theme 3 relates to primary reasons the nurses activated the RRT: abnormal clinical assessment finding; feeling worried; and an unmanageable patient condition. These were identified as appropriate reasons for activation (whether they did so or not); notably, the nurses commented this could vary depending on who the nurse was speaking to, which seemed to create confusion. The participating nurses named the RRT activation criteria; notably, these criteria are displayed at the study site, specifically abnormal (objective) physical assessment findings (i.e., decreased level of consciousness, new bradycardia or tachycardia, difficulty oxygenating, and symptomatic low or high blood pressures). A subjective feeling of worry about the patient's condition, with or without any supporting abnormal objective findings, also warranted RRT activation. Jessica shared, *so even though the vitals were looking okay, so we couldn’t really attribute it to that, it was much more underlying causes…which made me think I have to keep an eye on her because I’m not sure I can attribute it to what the team thought was going on.* Feeling unable to manage the patient on the unit with the staff and resources available was another reason to activate the RRT. Jessica elaborated: *the fact that things were getting too hard for me to handle and for my colleagues as well…I realized that I needed to call RRT, on top of this, because I didn't feel that we were able to manage this without RRT*.

**Theme 4: It Made it Easier for Me to Call the Next Time: Past (Positive) Experiences with the RRT.** Nurses reported positive past experiences with the RRT, including feeling supported by them and their unquestioning dependability, made them more likely to use the service again. This theme differs from Theme 2, as it is directly related to the prior experiences with the RRT. Overall, the nurses reported a favourable attitude toward the RRT. Hannah said, *everyone has a pretty good attitude about the RRT…the experiences I’ve had with the RRT nurses are really good.* Laura added, *when something's going on I’m like, I need someone who has a bit of a better expertise in this section than I do, for sure I need some help right and it's a little bit less scary when you’ve got lots of other people in the room helping you.* Elizabeth also stated, *it's kind of a safety net for us, and we know that they’re there if we need them.* Nurses feel the concerns they have raised about patients are being heard and validated by the RRT and their decision to involve them. Stacy shared, *I’ve always felt RRT is very much, like they’ll back up the nurse's opinion, like if you feel you need RRT, they will help you out*. Nurses felt the RRT encouraged their call and to use them again, if needed. Jessica stated, *so when people* [RRT members] *say that was okay you called us, it made it easier for me to want to call the next time…it leaves it more open for the nurse to call*.

#### Barriers

**Theme 5: I’m Not Sure I Ever Learned Why to Call - Lack of Education about RRT.** Theme 5 is about lack of education (e.g., no information; only receiving information once during orientation; one reported once having a mock (simulated) code) and mixed messages the nurses received about the RRT, including when and how to activate it; accordingly, the nurses wished for more frequent, regular RRT training. Although the nurses indicated knowing typical reasons to activate the RRT, they also felt they needed more education about when to call, how to call, or what type of call [code 99 or RRT activation] to make. Jessica said, *I’m not really sure I ever learned why to call RRT…good reason was never really explained…I think it needs to be reinforced, like as to reasons why you could call.* Regardless of years of experience, most of what the nurses know and have learned about the RRT is from observing peers or participating in activations. Laura shared, *Yeah, basically amongst your peers is how you figure that one out*. Elizabeth added, *I’m learning from them, I don’t know if they’re actually doing it correctly, either*.

Nurses repeatedly talked about not knowing whether to call the RRT on their portable phone or to call the hospital switchboard to activate on the overhead speaker, as well as what to do if they just wanted to ask a question of the RRT nurse, and whether the call should be a “code 99” or a “RRT activation”. Laura discussed,

Sometimes I’m a bit like reluctant to call them because, you know…like is this really something that I need to call like a code 99 for or do I need to like activate RRT, but how do I do that without activating overhead, and like things like that, it just gets a bit, the communication part on like how to properly do it is not always clear. I just wanted to activate RRT…consult RRT…the [hospital switchboard operator] was like ‘well, we need to call a 99 overhead’…which was not really necessary at the time and I think I probably like used a lot of resources.

On occasion, some have been reprimanded because of ‘calling the wrong team’. Elizabeth shared, *sometimes we get kind of push back because we’ve activated things incorrectly, and I don’t know if that deters people from calling*.

Everyone agreed RRT education or “refreshers” should be, at minimum, annually. Suggested educational strategies included: online modules and or videos; in-person discussions or training sessions; mock codes or simulation scenarios with the RRT; sessions to heighten familiarity with the *crash* [emergency] cart; involving the unit's clinical educator to disseminate information through emails, posters, or meetings; and development and use of a flowsheet outlining activation steps. Jessica suggested,

What would be a facilitator honestly would be more education around the RRT…a unit discussion…I think it wouldn’t be a bad idea to like, even a couple times a year…this is the RRT team…they’re going to talk to you today…about when to call a RRT…and to never feel like you can’t call a RRT…

**Theme 6: The Fear Factor in Activating RRT.** Theme 6 is about the trepidation nurses have activating the RRT, specifically the fear of being negatively judged on their nursing knowledge, skills and abilities by their peers, particularly if they made the ‘wrong decision’ to activate it. Hannah said, *there's also like the, the fear of calling RRT and then them coming, and then it being something I could have managed* [laughs]*…I don't know if it's like a judgment thing like, because I'm a junior and there's like the stigma of like…like my critical thinking may not be as advanced as other people's critical thinking.* Stacy added, *I feel like it's almost embarrassing for someone if they would call inappropriately*. Nurses also reported questioning themselves and their own nursing judgement after activating the RRT.

**Theme 7: We Don’t Want to Cry Wolf - The Unwritten Rules About When to/Not to Activate the RRT.** Theme 7 is about particular beliefs nurses have as to when they should contact the RRT, such as not wanting to call if they were perceived as being too busy (especially with something that might be more important) for fear they would be disturbing the team. Laura's hesitated to call, revealing *well, I knew that they were busy with somebody else*. John echoed the sentiment, saying:

*Just knowing how busy the hospital is, knowing if the ICU is packed, if other floors are packed, then they might think that it might not be the best to call just because the RRT may be busy, also when you heard the overhead page…you’re aware if a RRT call is going on somewhere else and you don’t ever really know when that RRT call is resolved…maybe some nurses might be hesitant to call, in the back of their head they feel like oh this, there was a RRT called 20 min ago…the RRT nurse might be busy. I think that might be a barrier…but really…knowing when it's finished shouldn’t be an indicator of when to call*.

Further, Stacy stated, *we don’t want to be using the service inappropriately. We don’t want to cry wolf and have them come and be like, you guys call me again for this?*

**Theme 8: The Team Wasn’t Listening to Me - The Tension in Calling (or Not) RRT.** Theme 8 is about primary team interactions when a nurse determines a patient is deteriorating, and the resulting inhibiting or motivating effect on getting a response to such a concern. Typically, nurses try to involve the primary team (starting with the junior residents and if needed, escalating to more senior residents until potentially reaching the attending physician), but if their response is not suitable (e.g., the nurse's call not being returned in a timely manner or the nurse feeling they are not being listened to), the nurse will include the RRT in the patient's care. Stacy shared, *not having a response from the team that's responsible for that patient…like you wait a ‘reasonable’ amount of time for the situation…but if they were symptomatic and you kind of had that feeling and you weren’t getting the help you needed, then you would call it* [the RRT] *quicker.* They [the team] may feel as though they have the situation under control and not want the extra help.

The nurses also expressed, when the primary team is unable to reach a resolution for the patient about which the nurse feels comfortable, it is difficult for them to overstep their decision. Laura shared, *especially when your, the main doctor has already told you don’t do anything about it and then you’re like well, am I going behind your back here like, so, it can be hard sometimes for sure*. Jessica agreed, *that's really hard to do. That's really, really hard to do because you're basically saying that my nursing skills are more in tune then physician assessment, is kind of how I felt*.

**Theme 9: Calling Over the Loudspeaker to Them Coming with Their Cart—**

**RRT Activation is Overwhelming.** RRT activation causes overwhelming feelings. When the RRT is called to the unit, including the activation being heard over the loudspeaker system for the entire hospital to hear, it can seem intimidating and ‘a big commotion’, which may cause nurses to reconsider activating it. Jessica shared, …*through initiating the call, it going over the loudspeaker, it's just that whole like, feeling that you’ve taken people out of there, I don’t know, place because we don’t know where you come from.* Additionally, some nurses felt once the RRT is activated, they take complete control of patient care. Elizabeth stated, *I found I was very much in the backseat…they kind of took over, they were still listening to what we had to say and helping, like our input was still definitely important, but the RRT nurse was doing most of it.* This reduced control and involvement with patient care was a feeling they did not like.

**Theme 10: We’ll Just Bypass This and Go Straight to RRT—Varying Unit Cultures and Implication to the RRT.** There are varying RRT activation practices between different nursing groups and shift times. The nurses shared some colleagues would bypass the usual unit practices and contact the RRT first to avoid unwanted delays or confusion. Different practices are reported to exist between: different units; experienced versus junior staff; different “lines” (or regular teammates); different groups (e.g., the floating nurses compared to the nurses who have permeant units); and different shifts. Hannah said, *It can be pretty hectic because I don’t know anything about the unit or the patients or the staff.* Whereas Laura shared, *Oh, our floor, we’re actually like really good…they* [float nurses] *love to work with us, because we do everything as a team…like even before any codes are called, we already had like three nurses in there helping us.* Some nurses also shared they may not have their regular team residents working overnight or only the attending staff on call, prompting them to call the RRT first. Elizabeth acknowledged,

*Usually during the night it gets kind of, the attending, who's not even there…so I find RRT often gets the brunt of it…often the neuro, neuro-stroke are under* (another speciality team), *who don’t know the patients…so they are unfamiliar and don’t know what to do…so then they have to talk to their senior, which seemingly takes forever at times, and so then sometimes we’re like, well we’ll just bypass this and go straight to RRT*.

**Theme 11: Colleagues Can Hold You Back in Calling—Varied (Mis)Communication RRT Practices.** There was miscommunication about the RRT—by RRT members and non-RRT members—and varied communication practices, causing additional confusion and discouraging its use. As an example, calling the RRT to establish intravenous access was viewed as acceptable by some and inappropriate by others. Laura expressed,

*I don’t want to bother them if they’re doing emergency things and I’m like, can you come start an IV? But we’ve also had some staff on RRT say that they’re not supposed to being doing IV starts…and don’t call us for this…whereas like staff are like yes, call us for this, so there's defiantly some miscommunication*.

Further, some nurses have discouraged others from activating the RRT. Jessica said,

*Well even sometimes colleagues can hold you back in calling. I was really nervous calling a RRT on it anytime because* I wasn't comfortable with some of the colleagues I was working with who felt that there was more ways that they could handle things versus calling somebody else…I *think there was an instance once I that I felt I was questioned …overly, more than I think I would normally be questioned as to why I had to call that RRT…so, that made me feel a little bit like* *I, calling a RRT for the wrong reasons*.

Notably, other nurses felt encouraged by staff members who promote the use of the RRT as a resource. John said, *there's an attitude of gratefulness the RRT does exist on my floor…the nurses we have in charge right now, they almost really promote the use of the RRT…the nurse educators…they’re very big on letting us know the importance of activating RRT and activating them early.* Hannah also shared, [it] *would be brought up a lot in safety huddles by management, if we see a sign, call RRT right away if needed*.

## Overarching Theme: Self-Imposed Complexity of Deciding to Activate the RRT

**Figure 1.**
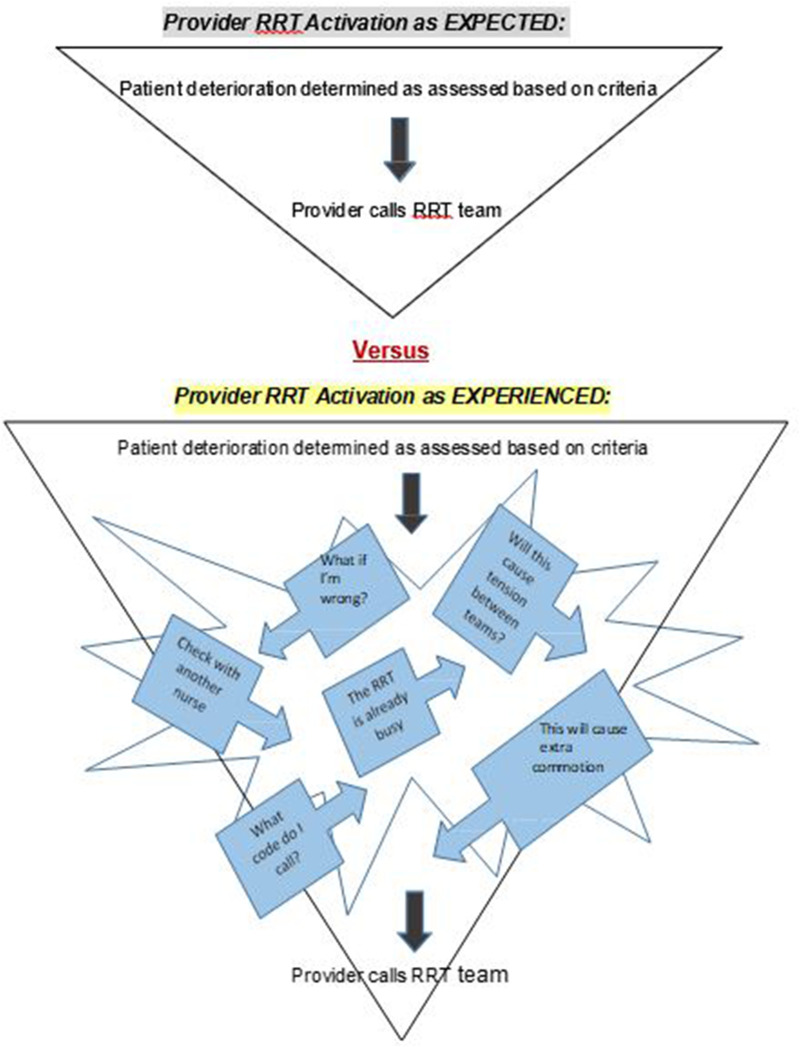
RRT Expected Activation versus RRT Experienced Activation.

In this study, it was illuminated nurses contemplate many factors (viewed as both positive and negative) in determining whether to activate the RRT—this cognitive processing is more complex than simply aligning with the clinical activation criteria. Once patient deterioration is detected, RRT activation is expected without delay; more realistically, however, there are several intervening considerations before the final decision to activate the RRT. These include issues such as worrying what others may think of oneself as a nurse, feeling anxiety about not wanting to bother the RRT if they are busy, and attempting to avoid unwanted stress between the teams. The overarching theme, *Self-Imposed Complexity of Deciding to Activate the RRT*, is about this internal dialogue nurses have while deciding whether to activate the RRT, which is multi-factorial and complicated. Patient safety is the priority, yet the findings bring to light, although the intentions of an RRT is to help prevent negative patient outcomes as early as possible, there is much more involved in an individual's decision to activate RRT—more than what is accounted for in policies or education (for unit nurses and RRTs). The implication of this complexity in decision-making is the potential delay in RRT activation, which is a safety concern. (Figure 1)

## Discussion

In this study was about the RRT activation on a general medicine unit, nurses shared occurrences from their work and offered recommendations and ideas about RRT education. Key considerations are in the following section.

### RRT Activation Criteria

In our study, the most common reason for activating the RRT is when a patient has abnormal, objective findings and has become unmanageable for the staff and their existing resources. This is in keeping with the nurses in Massey et al.'s (2014) interpretive qualitative study (interviews with 15 nurses), who reported activating the RRT for abnormal vital signs or ‘sensing’ a risk for deterioration. The nurses in our study, however, also indicated they did not always know why to activate the RRT (if it was perhaps not so obvious) or if their reason was deemed appropriate. In Shapiro et al.'s (2010) qualitative evaluation study with 56 nurses in the US, they also found nurses were uncertain as to when to activate the RRT, and the nurses had difficultly knowing the type of call to make or who needed to be called. Further, in our study, worry was described as a subjective feeling of concern for the patient and prompted nurses to seek further medical help; so too in Shapiro et al.'s (2010) study, the reasons nurses activated the RRT were objective signs and symptoms of deterioration and “worrying” about the patient. Raymond et al. (2019) found a nurse's worry often coincided with patient's actual progression to deterioration, warranting involvement by the RRT. Contrary to this, it has also been found subtle, subjective clinical changes caused hesitation and deliberation over RRT activation, as nurses felt the need for further justification, which was viewed as an activation barrier (Chua et al., 2019; Tilley & Spencer, 2020). In our study, these same feelings were shared by nurses, who had difficulty deciding if their concern was appropriate for RRT activation.

### Education About the RRT

The participants consistently requested more education about the RRT (including why and how to activate RRT [what type of call is needed for the situation]. The purpose, function, and policies of the RRT were also reported in the literature as unclear, and as such barriers to activating (Astroth et al., 2013; Chua et al., 2017; Chua et al., 2019; Jenkins et al., 2015; Massey et al., 2014; Moreira et al., 2018; Tilley & Spencer, 2020). Further, our study participants identified formal training about the RRT was rare, similar to another Ontario study (Johal, 2008). Further, most of our participants were educated on the job by observing or participating in the activations; this was troubling for some, as they felt unsure if what they were learning from others was correct. The nurses desired annual RRT training (at a minimum) via online modules, in-person training and hands on sessions (simulation). The importance of education in this regard cannot be overstated and is also reported as essential in variations of RRTs, such as psychiatric rapid response teams (Choi et al., 2019). In Choi et al.'s (2019) integrative reivew, they found that “[psychiatric] RRTs that included education, debriefing, and role modeling appeared to increase staff behavioral management skills and eventually reduced the need for RRTs” (p. 297).

### Communication Issues and the RRT

In this study, communication before (e.g., inability to reach the primary team), during (discussion and involvement) and after RRT activations (e.g., debriefs) varied, creating extra confusion and which may be a barrier to activating. Similarly, regarding communication prior to activation, Martland et al. (2016) reported the absence of effective communication increased anxiety and led to RRT activation. Related to debriefs, our study participants revealed they did not regularly occur (unless they initiated them), but it was something they desired. Aponte-Patel et al. (2018) reported nurses found group debriefing beneficial, helping improve RRT process understanding.

### Unit-Level Nurses Depend on Each Other for RRT Activation

Nurses in this study indicated they rely on their nursing peers for advice and support, including seeking help from those more experienced. They reported they first consult with each other, problem-solve, and perform tasks before activating the RRT. On the rare occasion, nurses felt “held back” from activating the RRT by an unfamiliar nursing colleague when they worked on a different unit that may have slightly dissimilar unit cultures and practices regarding RRT activation. Further, the years of nursing experience and increased confidence in one's skills and ability were mentioned as facilitators for activating the RRT. As well, the more times the nurse has activated the RRT, the more likely they were to do so in the future. These same findings are reported by Astroth et al. (2013), Halupa et al. (2018), Jenkins et al. (2015) and Padilla et al. (2018), concluding unit culture, leadership and support from coworkers, and past experiences with the RRT facilitate activation. Johal (2008) also described enhanced RRT activation occurred when there was knowledge sharing and collaboration between staff members. Notably, in Massey et al.'s (2014) study, they found consulting with peers about RRT activation was a facilitator and a barrier, as this was seen as delaying activation by taking extra time to deliberate, yet support was seen as a positive factor.

### The RRT Members

In this study, the overall attitude toward to the RRT was positive, and the participants felt supported by them. The nurses felt RRT members listened to their concerns and validated their reason for calling, as well as encouraged them to do so in the future. Based on other research, nurses have also positively described the RRT as supportive, validating, and an overall useful resource that modelled teamwork (Chua et al., 2017; Johal, 2008; Leach et al., 2010; McNeill et al., 2019; Menon et al., 2018). In Johal's (2008) quantitative study (*n* = 119 nurse participants from Ontario, Canada hospitals), open-ended questionnaire responses included evidence that suggested nurses’ beliefs about RRT activation was facilitated by: the guidance provided by the RRT; the RRT being viewed as a valuable resource; the RRT validating nurses’ concerns; previous (positive) experiences with the RRT; and the availability of the RRT and how quickly help is received. The Johal (2008) findings also suggested a positive but non-significant result the more often nurses used the RRT, the more they feel supported and knowledgeable. They associated this with positive learning outcomes for themselves and others regarding managing patient deterioration. In addition, strong interprofessional relationships were found to improve communication by Allen et al. (2017), resulting in earlier notice of clinical changes and more prompt RRT intervention.

### The Primary Medical Team and the RRT

The nurses in this study shared they tend to first call the most responsible member of the primary team before contacting the RRT. This is also indicated in other studies, in which there is the sociocultural belief nurses are to follow an inter-professional reporting hierarchy when escalating care (traditional model), of which the RRT is not considered a part (Allen et al., 2017; Bingham et al., 2020; Chua et al., 2017; Chua et al., 2019; McGaughey et al., 2017; Moreira et al., 2018; Tilley & Spencer, 2020). Notably, our study participants did not necessarily believe the RRT was not a part of this reporting hierarchy; rather that they first needed the patient's team involved in the care. As also reported in the literature, our participants said when they felt unsupported by the primary team, unable to contact them, or felt the patient was not being managed appropriately, they would activate the RRT for help (Chua et al., 2017; Chua et al., 2019; Padilla et al., 2018).

In this study, some nurses felt discouraged from activating the RRT by the primary team, perceiving the medical team felt they had the situation under control. In other related circumstances, they perceived the medical team did not feel trusted by the nurses, which resulted in nurse worry about tension between teams if they contacted the RRT. Johal (2008) also found the tension and conflict between the RRT and the primary team to be a barrier to activation, putting the nurses in difficult positions at times. Similarly, Chua et al. (2017) reported physicians felt activating the RRT implied their inability to manage the patient or was seen as a lost learning opportunity.

### Organizational Factors Affecting RRT Activation

Our study participants said they feel encouraged to use the RRT by those in management/leadership positions, such as charge nurses, educators, and managers. Formal RRT training at the hospital was very rare and mostly limited to only the hiring and orientation period. Nurses did report seeing posters about the RRT on their unit and had occasional casual discussions with their unit clinical educator; overall, however, they felt the training was inadequate and not nearly as frequent as they wished. Notably, Sebat et al. (2018) studied the effects of expanded administrative oversight of the RRT, which included strategies such as unit nurse RRT education and development of RRT protocols, and found it led to increased RRT activations.

### Fears Associated with Activating RRT

The study participants feared their nursing skills and abilities would be negatively judged by their peers and they would question themselves if they called for the RRT. They worried about activating the RRT for an “inappropriate” or “silly” reason, calling too frequently for minor patient concerns, or “bothering” the team if they were busy. Notably, these thoughts were not necessarily related to previous RRT activation incidents. Similarly, other researchers describe nurses’ fear of being criticized or reprimanded for inappropriate RRT activation (Chua et al., 2017; Chua et al., 2019; Currey et al., 2018; Massey et al., 2014; McGaughey et al., 2017; Menon et al., 2018; Padilla et al., 2018). In Astroth et al.'s (2013) generic qualitative study (semi-structured interviews with 15 medical-surgical nurses at one study site in the US), they also found the nurses hesitated to activate RRT out of fear their peers would think they did not know what to do, and so as not to bother the RRT if they were busy. In Massey et al.'s (2014) study, nurses described an RRT activation barrier as fear of causing extra “panic” on the unit, while the nurses in our study spoke of the overwhelming feelings of an RRT activation. Johal (2008) identified similar feelings of nurses regarding their increased stress on the unit once the RRT was present. Further, Johal (2008) identified some nurses felt relying on the RRT may result in loss of their skills and knowledge to manage deteriorating patients, which was a barrier to activation. Similarly, a participant in our study identified how a senior nursing colleague believed, since the introduction of the RRT, it reduced the chance for unit-level nurses to use their critical thinking skills as they had previously done. They felt this might also be the reason why some nurses hesitate to activate the RRT. They noted, with the RRT service, early identification and activation is essential but it is still an expectation nurses know what to do and how to manage patient deterioration (before the RRT arrives).

### Limitations

The study data was collected at a single site (hospital) over the summer of 2021 during the global COVID-19 pandemic. The logistics of staffing for summer vacations and limited unit manager coverage, combined with the stress of COVID-19, may have affected recruitment. As well, some participants recognized the first author (LF) as a RRT nurse, potentially affecting responses; to mitigate this, they were often reminded open, confidential dialogue was welcomed. Also, it may be that nurses hesitate to talk about the RRT if they do not feel knowledgeable about it and wish not to disclose that fact (including given the first author's (LF) professional role). Finally, the number of participants in this initial study was small and further research is needed. While there were notable repeated ideas and patterns from the participants, further research in varied geographic locations and hospitals and with participants of diverse gender and race is needed. Due to the scope, feasibility, and timelines of this study, the recruitment had to close. Nonetheless, these voices and this information is valuable and important to share for gaining a better understanding of unit-level nurses’ experiences with the RRT, particularly related to issues which have not yet been previously described in the literature.

### Nursing Education, Practice, and Research

This research has highlighted some educational opportunities for unit-level nurses and RRT members. From what the participants shared, there is not enough education about the RRT, and what is provided is not targeting the perceived barriers to activating. The participants offered some recommendations they believed would be helpful in improving the RRT activation process. Learning the appropriate reasons to activate the RRT and clarifying how to activate the RRT (i.e., who to call and what to say), were repeated learning needs. Some suggested strategies included: annual (at a minimum) online RRT training modules for unit-level nurses; in-person training from RRT members to help familiarize the unit staff with the RRT members; development of a pre-printed guide for the unit-level nurses to work through for each RRT activation; more posters displayed and or pocket-sized cards for the nurses to carry with information about the RRT; and more practice with mock codes or simulation training on RRT activations. Interdisciplinary RRT training to ensure the nursing and medical staff are getting consistent messages about the purposes of the RRT and reasons to activate is recommended. With respect to the knowledge gap in who to call and how to correctly word an RRT activation, regular training for the switchboard operators could also be implemented. Training for RRT members about how to lead a debrief post-activation would be beneficial and could possibly promote more frequent debriefing sessions—a wish expressed by the unit-level nurses. Finally, more interaction between the unit nurses and RRT members, such as informally and briefly visiting each unit each shift, as time permits. This may help to familiarize the unit nurses with the RRT members and form stronger working relationships, increasing the unit-level nurses’ comfort level with the team members and decreasing their level of apprehension to activating during patient deterioration scenarios. Finally, from a practice perspective, nurses’ perceived barriers to activating the RRT that are issues beyond the current training approaches in place. Further exploration is required to determine how best to address these issues before any practice changes are implemented and evaluated.

As this study was a descriptive, exploratory study, more future (Canadian) research is warranted, including across sites and with more diverse samples to broaden our evidence base. Further, in light of what was found in this study and the complexity of decision-making before RRT gets activated, it would be worthwhile to work with nurses in a *participatory action research approach* to further unpack this process and study what they determine needs to be actioned as a solution to reduce stigma and delay. For example, the question might be: “How can we improve RRT activation in our hospital community?”

## Conclusion

For the RRT team to reach its full potential, early recognition of patient deterioration and its prompt activation are vital to optimize favourable outcomes. This study was about nurses’ lived experiences on inpatient adult medicine units. Their narratives helped to illuminate RRT activation as an individual, nuanced, and complex decision-making process. For nurses, making this decision is not as simple as designed. For meaningful change regarding RRT activation, these practice complexities must be attended to in education and system improvements.
